# Causes of sudden neonatal mortality disclosed by autopsy and histopathological examination

**DOI:** 10.1097/MD.0000000000035933

**Published:** 2023-10-27

**Authors:** Doğuş Özdemir Kara

**Affiliations:** a Turkish Council of Forensic Medicine Ankara Head Office, Department of Pathology, Ankara, Turkey.

**Keywords:** autopsy, causes of death, histopathology, manner of death, neonatal

## Abstract

The neonatal period, or the first 28 days of life, is the most vulnerable time in a child’s life. Neonatal mortality has decreased in recent years. However, this progress varies at the national level, which necessitates actual regional data from different countries to identify local handicaps for life-saving precautions. This study aimed to investigate the causes for neonatal deaths as revealed by autopsy and histopathological examinations. A retrospective cross-sectional study was designed to identify the main causes of neonatal deaths in children who were autopsied at our institution between January 1, 2014, and December 31, 2021. Children who died within the first 28 days after birth (1–28 days of age) were referred to as neonatal cases. The main causes of neonatal death in children were determined via autopsy and histopathological and toxicological examinations. Furthermore, the causes of death were classified according to their manner of death. During this period, 122 neonatal children were autopsied at our institution. This group comprised 57 girls and 65 boys. For the manner of the death, natural causes were the most common cause (n = 91, 74.5%). Among natural causes, pneumonia (n = 66) was the leading one, representing 54% of all neonatal deaths, followed by perinatal conditions (n = 16, 13.1%). One of the pioneering reasons for death was sudden, unexpected postnatal collapse (n = 24, 19.6%), which was categorized under the undetermined group considering the manner of death. Unintentional (accidental) deaths accounted for 0.8% (n = 1) of total deaths, and intentional deaths were responsible for 6 neonates (4.9%) losses. This study shows that newborn children still die from simple and treatable infectious causes, probably arising from various familial and/or public inadequacies. In addition, sudden and unexpected postnatal collapse remains an important cause of neonatal mortality that has yet to be fully resolved. This study points out valuable inferences for caregivers and competent authorities to take preventive measures to prevent avoidable neonatal deaths.

## 1. Introduction

The neonatal period—the first 28 days of life—of childhood is the tenderest period of a child’s life. Children face the highest risk of dying in their first month of life, with an average global rate of 17 deaths per 1000 live births in 2019.^[1]^

In recent years, neonatal mortality has been decreasing,^[1,2]^ but this progress varies at the national level, necessitating actual regional data for different countries. Disclosing the main medical conditions, injury motives, and insults that affect neonates will help build protection strategies nationwide.^[3]^

According to a study published in 2017, Turkey achieved notable improvements, reaching targets of under-5-year mortality, neonatal mortality, and maternal mortality ratio between the years 2000 and 2016. Reducing the number of neonatal mortalities is an important part of the United Nations ‘Sustainable Development Goals.^[4]^ But child mortality rates are still high in low- and middle-income countries. Different studies have pointed to infectious motives as the leading cause of neonatal mortality.^[5]^

However, several other factors should also be considered. Injury-related deaths and undetermined cases (the ones for which the reason for death cannot be explained exactly) are concerning ones that we should also think about.

The objective of this retrospective cross-sectional study was to reveal the leading causes of neonatal mortality from our experience and to contribute to the development of intervention strategies in our country and around the world.

I believe that the data documented in this study are significant and problematic. These data reflect the burden of the most vulnerable demographics in society.

## 2. Material and methods

In this study, a retrospective cross-sectional study was designed to identify and classify the main causes of neonatal deaths in children who were autopsied at our institution between January 1, 2014, and December 31, 2021.

These autopsied neonatal children were those whose cause of death was unclear, sudden, unexpected, or problematic, according to public prosecutorship, which was the law’s competent authority for autopsy deaths.

All autopsies received by the Institute were handled in accordance with the medico-legal complete autopsy procedure. On the other hand, an autopsy started as a medico-legal autopsy by a forensic expert sometimes turned out to be a purely clinical autopsy for cases of sudden death. When ante-mortem efforts have failed to establish the cause of the death, widespread sampling of possible foci of infection is added to the procedure.

Data on deaths were obtained from the archives at our institution. Children who died within the first 28 days after birth (1–28 days old) were designated neonatal. In this study, only live births were included, and stillbirths were not mentioned.

The manner of death is classified based on the type of conditions that cause death and the circumstances under which they occur. The categories of the manner of death are: natural, unintentional (accident), intentional (homicide, suicide), and undetermined (or “could not be determined).^[6]^ The causes of death were classified according to this definition.

All injuries were classified according to the International Statistical Classification of Diseases and Related Health Problems, version 10 (World Health Organization).^[7]^ In this classification schema, unintentional injuries are identified as road injuries, poisoning, falls, fire, heat and hot substances, drowning, and exposure to mechanical forces. Intentional injuries include self-harm, interpersonal violence, collective violence, and legal intervention. Accidental threats to breathing (suffocation, choking, and strangulation) and complications of medical or surgical care cause other unintentional injuries.

This study was reviewed and approved by our review board and ethics committee (No. 21589509/2018/971) on December 25, 2018.

The data that support the findings of this study are available from our institution, but restrictions apply to the availability of these data, which were used under the license for the current study and so are not publicly available. However, data are available from the authors upon reasonable request and with permission from our institution.

During the study, care was taken not to violate the principle of confidentiality and to protect the personal or institutional data obtained from the subjects. In accordance with the ethical rules, the identities of the subjects involved in the study were kept confidential.

Analyses were performed using Statistical Package for the Social Sciences Version 29.0 (IBM SPSS Statistics for MacOS, Version 29.0, Armonk, NY). Nominal variables were reported as n (percentages) and compared using a two-tailed Chi-square. The *P* value was set at <.05 for statistical significance.

## 3. Results

This study covers 8 years, from 2014 to 2021. In a period of 8 years, a total of 122 neonatal autopsies were performed at the institute, and since this is the only authorized institution in Ankara where neonatal autopsies are performed, the total number of neonatal autopsies performed during this period is 122.

According to data from the Turkish Statistical Institution, 4119 neonatal deaths occurred in Ankara between 2014 and 2021.^[8]^ As a result, approximately 0.02% of neonatals underwent autopsies during this time interval.

The study group consisted of 57 girls and 65 boys. Seventy-four of the children came from the city center and 48 from small towns.

Natural deaths were responsible for 74.5% (n = 91) of total deaths. The most common cause of natural death was pneumonia (n = 66), representing 54% of all neonatal deaths. These children did not have any prior medical conditions but undiagnosed and untreated pneumonia, which was diagnosed after autopsy via microscopic examination of the lungs by a pathologist.

Other significant natural reasons were perinatal conditions, causing (n = 16) 13.1% of all the neonatal deaths. Documented “perinatal conditions” included premature birth and respiratory distress syndrome (RDS) (n = 7), umbilical cord entanglement (n = 1), meconium aspiration (n = 6), and perinatal hypoxic-ischemic encephalopathy (n = 2). In the “perinatal condition” group, some children also had pneumonia. However, as in this group, pneumonia was secondary to their primary medical condition, and they were classified under the “perinatal conditions” group.

Three children in the natural death group had fatal congenital heart diseases: 1 died of myocarditis, 1 died of diarrhea, and 1 died of complications of metabolic disorders. Twenty-four (19.6%) cases were found dead in their cribs. Any medical reason or any direct force to explain their deaths could not be found. Microscopic examination of the lungs revealed that 9 of these children had aspirated human milk or baby formula. These cases were classified as sudden and unexpected postnatal collapse (SUPC group, which contained all-suspected accidental suffocation and strangulation in bed and an ill-defined cause of death). Considering the manner of death, these cases were categorized into an undetermined group.

Unintentional (accidental) deaths accounted for 0.8% (n = 1) of total deaths. The child died of severe burns after being trapped in a house fire. As intentional deaths were evaluated (n = 6, 4.9%), one newborn was abandoned to die in a trash bin, and 5 were murdered (1 penetrating, 4 blunt trauma), summing up to 6 children being abuse victims.

Causes of death according to the manner are summarized in Table 1.

**Table 1:** The causes and the manners of death for neonatal children.

Manner of death	Cause of death	n	Total	%
Natural	Pneumonia	66	91	74.59
	Prematurity + RDS + meconyum aspiration	13		
	Prenatal hypoxic ischemic encephalopathy	2		
	Umblical cord entanglement	1		
	Congenital heart disease/myocarditis	4		
	Congenital metabolic disorders	1		
	Congenital diaphragma agenesia	1		
	Congenital hypoplastic lung	1		
	Intestinal invagination	1		
	Enteritis	1		
Undetermined	Sudden unexpected postnatal collapse	24	24	19.67
Unintentional	Thermal injury	1	1	0.81
Intentional (homocide)	Blunt trauma	2	6	4.91
	Penetrating trauma	2		
	Abandoning	2		
		Total	122	100

The results of the chi-square analysis examining the relationship between the number of deaths of the decedents according to years, gender, and death manners are given in Table 2. When the table is analyzed, it is determined that there is no statistical relationship between the number of decedents according to years and gender (*P* = .753) and death manners (*P* = .603).

**Table 2:** Manner and gender distribution of the deaths by year.

	Years n (%)								*P* value
	2014 (n = 19)	2015 (n = 21)	2016 (n = 16)	2017 (n = 15)	2018 (n = 17)	2019 (n = 11)	2020 (n = 11)	2021 (n = 12)	
*Gender*									.632
Male	10 (52.6)	9 (42.9)	9 (56.3)	8 (53.3)	8 (47.1)	9 (81.8)	5 (45.5)	7 (58.3)	
Female	9 (47.4)	12 (57.1)	7 (43.8)	7 (46.7)	9 (52.9)	2 (18.2)	6 (54.5)	5 (41.7)	
*Death*									.878
Intentional	1 (5.3)	1 (4.8)	0 (0)	1 (6.7)	1 (5.9)	1 (9.1)	0 (0)	1 (8.3)	
Natural	16 (84.2)	18 (85.7)	10 (62.5)	11 (73.3)	13 (76.5)	7 (63.6)	8 (72.7)	8 (66.7)	
Undetermined	2 (10.5)	2 (9.5)	5 (31.3)	3 (20)	3 (17.6)	3 (27.3)	3 (27.3)	3 (25)	
Unintentional	0 (0)	0 (0)	1 (6.3)	0 (0)	0 (0)	0 (0)	0 (0)	0 (0)	

The distribution of the number of deaths over the years and the main causes of death for each case are presented in Table 3. When the table is analyzed, it is determined that there is no statistically significant relationship between the number of deaths in years and the main causes of death of the decedents (*P* = .332).

**Table 3:** Distribution of main causes of the deaths by year.

	Years				*P* value
	2014–2015 (n = 35)	2016–2017 (n = 27)	2018–2019 (n = 24)	2020–2021 (n = 20)	
*Causes of death*					.332
Pneumonia	24 (68.6)	14 (51.9)	17 (70.8)	11 (55)	
SUPC*	4 (11.4)	8 (29.6)	6 (25)	6 (30)	
Perinatal complication	7 (20)	5 (18.5)	1 (4.2)	3 (15)	

* Sudden unexpected postnatal collapse.

## 4. Discussion

The neonatal period covers the first 28 days after birth. The most frequently reported causes of neonatal deaths are infections, perinatal asphyxia, metabolic diseases, and congenital malformations.^[9–12]^

In this study, natural deaths were responsible for 74.5% (n = 91) of total deaths. As a natural cause, the most common one in the group was pneumonia (n = 66), representing 54% of all the neonatal deaths. These decedents had no other preexisting medical conditions. The etiologies of these pneumonia cases were viral and/or bacterial infections.

Pneumonia is the leading cause of child mortality, causing between 152.000 and 490.000 infants to die before the age of one. This is a serious global health burden for developing countries. Immature lung immunity, nonspecific and usually subtle symptoms, and the rapidly advancing nature of infection make it difficult for parents to recognize and take action, which is an important task for clinicians.^[13]^ Caregivers must be informed by professionals about signs, particularly of new couples who become parents for the first time at an early age. Overall, a significant proportion of neonatal deaths due to pneumonia occur in developing countries, and the main cause of community-acquired pneumonia is Streptococcus pneumoniae, and the main viral cause is respiratory syncytial virus.^[14]^

According to my study’s findings, perinatal conditions (n = 16) (premature birth and RDS, umbilical cord entanglement, meconium aspiration syndrome, and prenatal hypoxic-ischemic encephalopathy) were significant factors responsible for 13.1% of neonatal deaths.

Precise infections, asphyxia, and prematurity are perinatal-related events that are important contributors to neonatal death, especially in developing regions of the world.^[15]^ As it has been practiced in high-income countries, applying antenatal and perinatal controls and determining critical cases to refer them to specialized pediatric clinics is essential. This is because the time a newborn loses is priceless and too costly for both the baby and society. Taking simple steps, such as contraception, vaccination of pregnant women, hygienic delivery at the hospital, and training healthcare workers in resuscitation, will reduce preventable deaths in the neonatal period.^[10]^

The second leading cause of death in this group was “sudden unexpected postnatal collapse of the newborn”, as called sudden unexpected death in early infancy (n = 24), representing 19.6% of all deaths. Considering the manner of death, these cases were categorized into an undetermined group. The children in this study did not have any prior medical problems and mostly shared the same story of being found dead in bed. No signs of trauma or toxic substances were observed during the screening tests.

In this study, histopathological examination showed that 9 children had aspirated breast milk or baby formula without any signs of pneumonia. In the SUPC group, evidence of physiological stress in the thymus and/or lungs was detected, expressed as hemorrhage. Although there was no clear evidence of any medical problem, some decedents had a history of prodromal illness. Bed sharing, heavy lungs, and deaths during sleep were other features defined by some authors, and I also detected them in this study.^[16]^

Approximately one-third of the SUPC events occur during the first week of the neonatal period. Mild gliosis of the brainstem, which controls the cardiorespiratory system, has been observed in some cases. These authors suggest that any insult caused by ischemia in these areas or immaturity can make these newborns vulnerable to known risk factors for SUPC.^[17]^ Though any gliosis was not observed via microscopic examination, in my study, some decedents were found to have white matter gliosis during autopsy.

A study from the United States revealed low levels of butyrylcholinesterase-specific activity in the blood of decedents who were classified as sudden unexpected death in infancy deaths. As butyrylcholinesterase is an enzyme of the cholinergic system, they suggest that it can be a good predictive measure of the autonomic (dis)function of the newborn, which may lead to a specific vulnerability to their death.^[18]^ This vulnerable phase must be well explained to caregivers to raise awareness about the risks, and close surveillance of newborns should be encouraged.

Totally, accidental deaths accounted for 0.8% (n = 1) of all deaths. One child died of severe burns after being trapped in a house. In this study, intentional deaths were (n = 6) 4.9% of all deaths. Intentional insult toward children is, unfortunately, a mutual problem for many countries and mostly remains undiscovered, and the real numbers of fatal ones are unknown.^[19]^ Neonaticide (killing an infant shortly after birth, usually on the first day of life) and infanticide (the murder of a child aged 1 day to 1 year) are generally performed by the victim’s mother.^[20]^

A systematic review of the worldwide incidence of neonaticides in Europe found that young maternal age, being unmarried, and primiparous are common features shared by these perpetrators.^[21]^

One study from Turkey about the sociodemographics of mothers who abandoned their newborn babies at the hospital also found that, low education level, being unmarried, and/or being unemployed were the most important grounds shared by mothers.^[22]^

Another study from the United States pointed out racial/ethnic disparities as a disadvantage for victims. Studies have shown that male and non-Hispanic blacks are more likely to be victims of infanticide and neonatal deaths.^[23]^

According to data from the Turkish Statistical Institute, the rates of neonatal deaths decreased over the years from 7.3 in 2014 to 6 in 2021.^[24]^ Looking at ‘United Nations International Children’s Emergency Found’s neonatal mortality statistics for Turkey, although the number of deaths by year does not exactly overlap with the numbers of the Turkish statistical institution, similarly, there has been a decrease in the rate of neonatal deaths over the years, from 7 in 2014 to 4 in 2021.^[25]^ In proportion to this, the number of neonatal cases autopsied in our institution has decreased over the years (Table 4).

**Table 4:** Number of cases and manner of deaths by years.

	Years				*P* value
	2014–2015 (n = 40)	2016–2017 (n = 31)	2018–2019 (n = 28)	2020–2021 (n = 23)	
*Gender*					.753
Male	19 (47.5)	17 (54.8)	17 (60.7)	12 (52.2)	
Female	21 (52.5)	14 (45.2)	11 (39.3)	11 (47.8)	
*Death*					.603
Intentional	2 (5.0)	1 (3.2)	2 (7.1)	1 (4.3)	
Natural	34 (85.0)	21 (67.7)	20 (71.4)	16 (69.6)	
Undetermined	4 (10.0)	8 (25.8)	6 (21.4)	6 (26.1)	
Unintentional	0 (0)	1 (3.2)	0 (0)	0 (0)	

Unfortunately, the causes of neonatal deaths are not detailed in the statistics of the Turkish Ministry of Health or in the statistics of the Turkish Statistical Institute, which increases the value of this study.^[26,27]^

In addition, as in many countries, the “underlying cause of death” used in the classification of infant mortality in Turkey is not compatible with the International Statistical Classification of Diseases and Related Health Problems, version 10 classification established by World Health Organization. Therefore, the results of the Ministry of Health and the Institute of Statistics are not reliable. However, since the information provided in both sources includes the neonatal period within the infant period, only the records of the neonatal period could be accessed. For this reason, the results of the study could not be compared with the causes of neonatal mortality in Turkey.

There are only a few studies searching for the causes of neonatal mortality in Turkey. In a study on neonatal mortality in Turkey, when evaluated together with infant mortality, the most common causes of death were: prematurity, congenital anomalies, congenital heart diseases, sepsis and perinatal asphyxia, pneumonia, RDS, and sudden unexpected death in infancy.^[28]^

In a 2018 study on infant mortality conducted in Adana, sepsis was found to be the most common cause of death, followed by prematurity and problems related to prematurity (12.6%), congenital heart disease and complications (12.6%), respiratory problems (11.9%), congenital anomalies (9.8%), immaturity (<750 g birth weight or 24 gestational weeks) (8.4%), and sudden death (6.0%).^[29]^

In another study conducted in Bursa, when the causes of neonatal deaths between 2010 and 2012 were analyzed, 68.2% of infant deaths occurred in the newborn period. The main causes of infant deaths were prematurity (36.3%), congenital malformations and chromosomal diseases (34.3%), perinatal causes (12.9%), and sudden infant death syndrome (6.2%).^[30]^

In all of the above studies, the neonatal period is expressed as mixed with infant demise. Since the present study was a postmortem study, the number of deaths due to pneumonia was much higher than in the above hospital studies. Most likely, these cases in my study represent cases that somehow went unnoticed by parents and doctors, so eventually their deaths ended up being judged problematic by the prosecutors.

The rate of cases of sudden, unexpected neonatal collapse was high in my study compared to hospital studies, as these cases’ deaths were unexplained and they were frequently referred for autopsy.

A verbal and social autopsy in Indonesia found that the most common causes of neonatal death were prematurity and perinatal complications. However, as this was a verbal autopsy, no further diagnosis could be made.^[31]^

Another verbal autopsy study from the United States reported that delays in access to health care were closely associated with neonatal death but did not elaborate on the causes of death.^[32]^ As a pure hypothesis, inadequate access to health care may also contribute to the high infectious-related mortality in the present study.

In a verbal autopsy study from Kenya, perinatal asphyxia in the early period and infectious causes in the late period were found to be the most common causes of neonatal death.^[33]^ In the present study, since the number of cases fitting the early period was very few (n = 7), the causes were not analyzed as early or late periods.

Globally, sub-Saharan Africa has the highest rate of neonatal mortality and accounts for 38% of global neonatal deaths.^[34]^ In a verbal autopsy study conducted in Ghana in this region, asphyxia and prematurity were found to be the most common causes of neonatal death.

In low- and middle-income countries, verbal autopsy is performed instead of full diagnostic autopsy due to infrastructural deficiencies, religious, and cultural reasons, and its reliability is limited.^[35]^

In a neonatal autopsy study conducted in Brazil, postmortem pathological diagnosis added 34% new findings to the neonatal specialist diagnosis; in 6.9% of cases, relatives were referred to genetic counseling; and in 9.6% of cases, results were reached that would have changed the treatment modality if caught in the premortem stage.^[36]^

Therefore, this study, which provides complete and diagnostic autopsy results, is a good contribution to the literature.

This study centers only on uncertain, sudden, unexpected, or problematic neonatal deaths. This may lead to a selection bias as it may not be representative of all neonatal deaths. Such a focus may distort the results towards specific causes of death that are more likely to be uncertain or sudden, potentially limiting the generalizability of the findings to the wider population of neonatal deaths. Nevertheless, this feature of the study has revealed topics that are overlooked or underemphasized in the clinic. For instance, the rate of pneumonia in the presented study, which is higher than other studies conducted in hospitals, shows our colleagues what we may have overlooked. Also, since all SUPC cases were autopsied, the study reminds our colleagues once again of the reality of SUPC, which is not often thought of in the clinic.

## 5. Conclusions

This study shows that newborn children still die from simple and treatable infectious causes, probably arising from various familial and/or public inadequacies. Health services should be made more accessible and widespread during a vulnerable period, such as the newborn. Besides, colleagues should be more alert about neonatal pneumonia and consider hospitalizing and monitoring patients closely.

In addition, SUPC remains an important cause of neonatal mortality that has yet to be fully resolved. Therefore, this gentle period should be properly explained to parents during their doctor visits, and close surveillance of the baby should be encouraged.

I believe that this study points out valuable inferences for caregivers and competent authorities to take preventive measures to prevent avoidable neonatal deaths.

In my opinion, larger case series are also needed to explain more details.

This study did not receive any specific grants from funding agencies in the public, commercial, or not-for-profit sectors.

## Author contributions

**Conceptualization:** Doğuş Özdemir Kara.

**Methodology:** Doğuş Özdemir Kara.

**Project administration:** Doğuş Özdemir Kara.

**Writing – original draft:** Doğuş Özdemir Kara.

**Writing – review & editing:** Doğuş Özdemir Kara.
